# The protective effects of Crocin on testopathy in fat-fed and streptozotocin-treated diabetic rats: An experimental study

**DOI:** 10.18502/ijrm.v17i2.3986

**Published:** 2019-03-20

**Authors:** Saeed Mirzaee, Mohammad Ehsan Bayatpoor, Shima Shahyad, Mohammad Taghi Mohammadi, Zahra Bahari

**Affiliations:** ^1^Students' Research Committee, Baqiyatallah University of Medical Sciences, Tehran, Iran.; ^2^Neuroscience Research Center, Baqiyatallah University of Medical Sciences, Tehran, Iran.; ^3^Department of Physiology and Medical Physics, Faculty of Medicine, Baqiyatallah University of Medical Sciences, Tehran, Iran.

**Keywords:** *Crocin*, *Reproductive system*, *Blood glucose*, *Streptozotocin*, *Diabetes.*

## Abstract

**Background:**

Male hypogonadism is associated with type II diabetes mellitus due to testicular dysfunction. Medicinal plants have received considerable attention for the management of diabetes and its complications.

**Objective:**

The aim of present study was to evaluate the anti-diabetic and protective influence of Crocin on testopathy in diabetic rats.

**Materials and Methods:**

In this experimental study, type II of diabetes mellitus was induced by high-fat diet and low dose of streptozotocin. Male Wistar rats (8 weeks, 150–200 gr, 18 rats; *n*= 6 per group) were divided into a control group (standard diet), diabetic group (streptozotocin+high-fat diet), and treatment group (High-fat diet+streptozotocin+Crocin at 20 mg/kg/day, i.p. for 60 days). After 60 days, animals were euthanized, testis and epididymis were dissected, and weights of testes and sperm count were analyzed. Hematoxylin-eosin-stained was done for histopathological examination. Blood samples were collected for the assessment of serum glucose and cholesterol.

**Results:**

High-fat diet and streptozotocin significantly increased the serum glucose and cholesterol levels as compared to the control group (p≤ 0. 001). Moreover, there was a significant decrease in the weight of right (p= 0.008) and left testes (p≤ 0. 001) and also the total sperm count (p= 0.023) in the diabetic group compared with the control group. Current results also identified that type II diabetes mellitus induced degeneration in the morphology of seminiferous tubules. Application of Crocin could significantly decrease serum glucose and cholesterol levels (p= 0.003). Furthermore, Crocin treatment significantly increased the weight of the right (p= 0.026) and left (p= 0.014) testes and total sperm count (p= 0.000). Also, Crocin could attenuate the pathological changes of the testes in the treatment group.

**Conclusion:**

Present findings concluded that Crocin treatment improved diabetic testopathy and impairment of seminiferous tubules induced by high-fat diet and streptozotocin.

## 1. Introduction

Diabetes mellitus (DM) leads to metabolic abnormalities involving regulation of carbohydrate, lipids, and proteins metabolism. These abnormalities produce long-lasting complication in a variety of organs (1). Disturbance in the male reproductive system is associated with type II diabetes mellitus (DMII) due to testicular dysfunction (2, 3). Glucose metabolism is critical for spermatogenesis and also specific functions of mature sperm such as motility and fertilization ability (4). Ample pieces of evidence have shown that DM decreases testicular weight, sperm count, motility of sperm, and also increases the abnormal sperm in the testes (5, 6). Multiple factors are involved in the onset of diabetic pathology. It seems that chronic hyperglycemia and oxidative stress are playing a critical contribution in the pathogenesis of DM (7, 8). In this context, medicinal plants have received considerable attention for the management of diabetes and its complications, because of their antioxidant properties (9, 10). Crocin, the extracts of Crocus sativus stigmas, identified as an antioxidant agent (11, 12). This phytochemical compound exerts many pharmacological effects including antioxidant and anti-inflammatory (13), antinociceptive (14, 15), and neuroprotective (16–19) activities. Animal models have played an important role in the characterization of disease pathophysiology and identification of novel therapeutic avenue. The streptozotocin (STZ) model of diabetes is commonly used for better understanding of antidiabetic drugs (20, 21). However, STZ caused significant destruction of the pancreatic mass and may therefore mimic changes closer to type I diabetes (DMI) rather than DMII. High-fat diet (HFD) might change energy metabolism, insulin resistance, and dyslipidemia in rodents and humans (22). Some study reported that the combination of HFD with STZ has successfully mimicked natural progress of diabetes development in human DMII (23–25).

Therefore, the present study was designed to evaluate the protective effects of Crocin on male reproductive dysfunction induced by HFD and STZ in rats. In the current study, we also estimated the effects of Crocin on sperm count, testis weight, and histopathological changes of testis. To the best of our knowledge, this is the first study to evaluate the protective effects of Crocin on male testopathy in DMII induced by HFD/STZ in rats.

## 2. Materials and Methods

### Animals

Adult male Wistar rats (weighing 150–200 gr, eight weeks) were used in this study. Animals were kept under standard laboratory conditions (temperature: 25±2${}^{\circ }$C; humidity: 60±5%; 12 hr light dark cycle, and free access to water and standard diet). After a one-week accommodation period, animals were randomly divided into three groups (*n*= 6 per group). These groups were as follows: group 1: control group (intact and standard diet); group 2: diabetic group (DMII) (STZ+cholesterol diet containing 4% cholesterol); and group 3: treatment group (Crocin+STZ+cholesterol diet). The animals with blood glucose concentration of > 250 mg/dl were used for the study. Crocin (20 mg/kg/day) (26) was simultaneously administered intraperitoneally in the treatment group from the beginning of the study for 60 days. At the termination of the test, serum glucose and cholesterol levels, sperm count and testis weight were evaluated. Moreover, hematoxylin and eosin were used for staining of testis tissue in all groups.

### Chemicals

Crocin [digentiobiosyl all-tarnscrocetin‎ (8, 8'-di-apocarotene-8, 8'-dioic acid) ester] and STZ were purchased from Sigma–Aldrich Inc. (St Louis, MO, USA). Crocin and STZ were dissolved in normal saline.

### Induction of diabetes

DMII was induced in overnight-fasted rats by HFD (4% of cholesterol) and a single intravenous (i.v.) injection of STZ (40 mg/kg of body weight) into the tail vein of animals lightly anaesthetized with ether (27). STZ was dissolved in normal saline. Animals were fed HFD for 60 days. The glucose level and insulin level of the same rat were measured 72 hr after the injection of STZ. The animals with blood glucose concentration of > 250 mg/dl were used for the study. We also measured the level of blood glucose at the end of the experiment (day 60 of the experiment).

### Serum glucose and cholesterol determination

Samples were collected from tail vein; plasma was separated by centrifuge at 3000×g for 10 min. Serum samples were collected and stored at -20${}^{\circ }$C until analysis. Glucose level was estimated by glucose enzymatic kit using spectrophotometer on day 60, after the induction of diabetes. Blood glucose was measured using enzymatic colorimetric method (28) and cholesterol by the method of Zlatkis on day 60, after the induction of diabetes (29).

### Surgical procedure

Animals were sacrificed using diethyl ether anesthesia and laparotomy was performed on the 60th day of present study. Sample tissues (testes and epididymis) were removed in all rats. The left epididymis tissues were used for the evaluation of sperm counts. The weight of testes (right and left) was evaluated. Furthermore, for histological estimations, the left testis was processed using hematoxylin and eosin (H and E) staining method (30).

### Collection of spermatozoa for the evaluation of sperm count

For sperm counting, left epididymis (the cauda part) was cut into small pieces and incubated in pre-warm normal saline (10 mL) at 37${}^{\circ }$C. Sperm was gently forced out of the cauda epididymis with fine forceps. Sperms were counted using Neubauer chamber cell counting under 10 magnifications by optical microscope (30).

### Histopathological examination

Samples analyzed by H and E method (31). Formalin fixed testis samples embedded in paraffin. Then, samples were sectioned at five-micron and stained by H and E. The tissue sections were analyzed under light microscopy using Johnsen's criteria, scoring 1–10 (31). Score 1 show no germ or Sertoli cells in tubular section. Score 2 show present of only Sertoli cells with no germ cells. Score 3 show only spermatogonia present. Score 4 is only a few spermatocytes with no spermatozoa. Score 5 show no spermatozoa or spermatids present but many spermatocytes. Score 6 is only a few spermatids present. Score 7 show present of spermatozoa and many spermatids. Score 8 show only a few spermatozoa present. Score 9 show present of many spermatozoa but germinal epithelium disorganized. Score 10 means perfect tubules and present of many spermatozoa.

### Ethical consideration

The study was conducted in accordance with the Guidelines of the National Institute of Health (NIH) for the Care and Use of Laboratory Animals, and was approved by the local ethical committee (The Baqiyatallah University of Medical Sciences Committee on the Use and Care of Animals, IR.BMSU.REC.1396.174).

### Statistical analysis

Statistical analyses were performed with the SPSS software (Statistical Package for the Social Sciences, version 21.0, SPSS Inc, Chicago, Illinois, USA). Data are expressed as mean±SEM. Multiple groups were compared using one-way analysis of variance followed by the Tukey HSD post hoc comparisons, and p< 0.05 was considered significant.

## 3. Results

### Blood glucose and cholesterol levels

**Figure 1:**
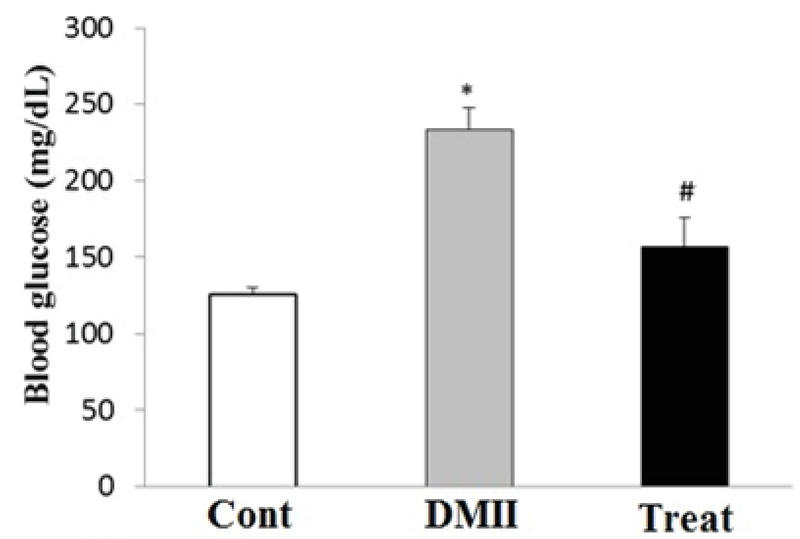
The level of serum glucose in control and experimental groups. Data are presented as mean±SE. Serum glucose levels was evaluated on 60 days after the induction of DMII. ${}^{*}$p< 0.05 indicates the significant differences with control group; ${}^{\#}$p< 0.05 indicates the significant differences with DMII group; Cont: Control group; DMII: Diabetic group; and treat: Treatment group.

**Figure 2:**
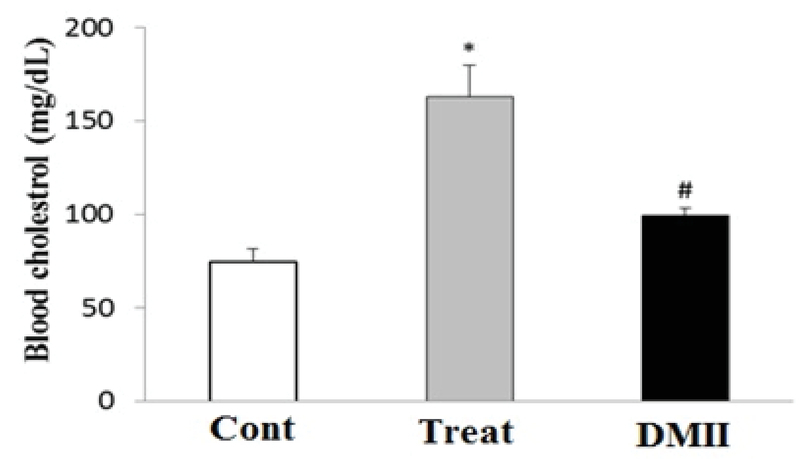
The level of serum cholesterol control and experimental groups. Data are presented as mean±SE. Serum cholesterol levels was evaluated on 60 days after induction of DMII. ${}^{*}$p< 0.05 indicates the significant differences with control group; ${}^{\#}$p< 0.05 indicates the significant differences with DMII group; Cont: Control group; DMII: Diabetic group; and treat: Treatment group.

Figures 1 and 2 show the levels of serum glucose and cholesterol in control and experimental groups, respectively. There was a significant elevation in serum glucose and cholesterol levels in DM group as compared with the control group (p≤ 0. 001). Furthermore, our results identified that the application of Crocin significantly decreased glucose (p= 0.003) and cholesterol (p= 0.003) levels in the treatment group as compared with the DM group. There was no significant difference in glucose and cholesterol levels between the control and treatment groups (Figure 1, 2).

### Sperm count

**Figure 3:**
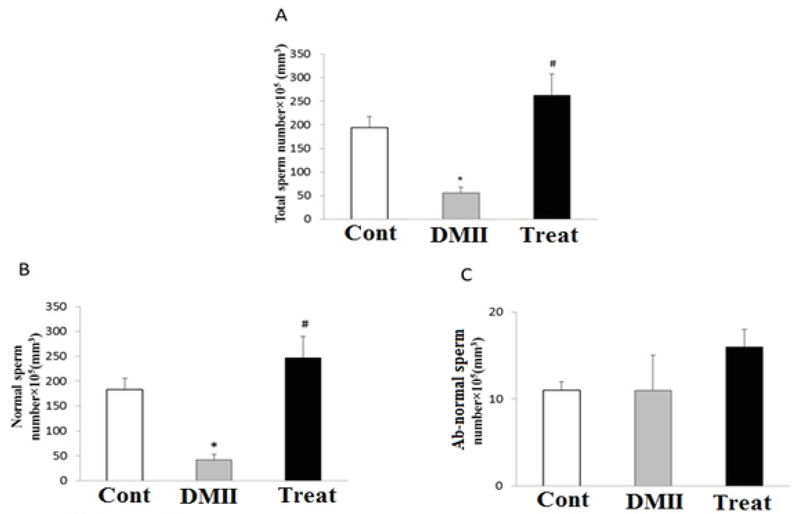
Effects of application of Crocin on (A) total sperm number, (B) normal sperm number; and (C) abnormal sperm number in control and experimental groups. Data are presented as mean±SE. ${}^{*}$p< 0.05 indicates the significant differences with control group; ${}^{\#}$p< 0.05 indicates the significant differences with DMII group; Cont: Control group; DMII: Diabetic group; and treat: Treatment group.

STZ/HFD treatment significantly decreased the total sperm count (Figure 3(A), p= 0.023) and the number of normal sperms (Figure 3(B), p= 0.024) in the diabetic group compared with the control group. Furthermore, our data identified that the application of Crocin significantly increased the total sperm count (Figure 3(A), p= 0.000) and also the number of normal sperms (Figure 3(B), p= 0.000) in the treatment group as compared with the diabetic group. There was no significant difference in the total sperm count and the number of normal sperms between the control and treatment groups (Figures 3(A), (B)). Administration of Crocin could not have any significant effect on the number of ab-normal sperm in treatment group as compared with the diabetic group (Figure 3(C)).

### Testis weight

**Figure 4:**
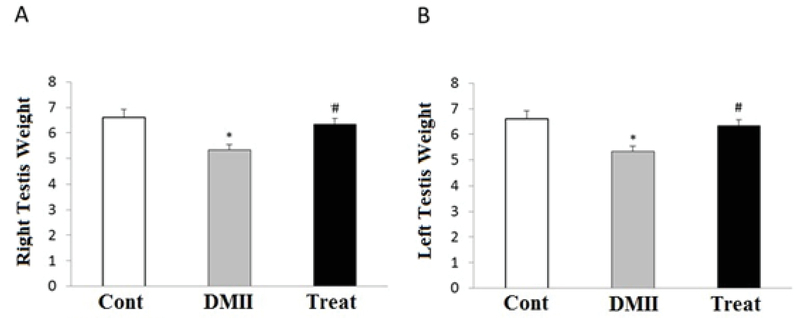
Effects of application of Crocin on testis weight alteration induced by HFD/STZ in control and experimental groups. ${}^{*}$p< 0.05 indicates the significant differences with control (cont) group; ${}^{\#}$p< 0.05 indicates the significant differences with DMII group; Cont: Control group; DMII: Diabetic group; and treat: Treatment group.

As shown in Figure 4, there was a significant decrease in the weight of right (p= 0.008) and left (p≤ 0. 001) testes in the diabetic group compared with the control group. Furthermore, the application of Crocin significantly increased the weight of the right (p= 0.026) and left (p= 0.014) testes in the treatment group compared to the diabetic group (Figure 4). There was no significant difference between the control and treatment groups (Figures 4(A), (B)).

### Histopathological finding

**Figure 5:**
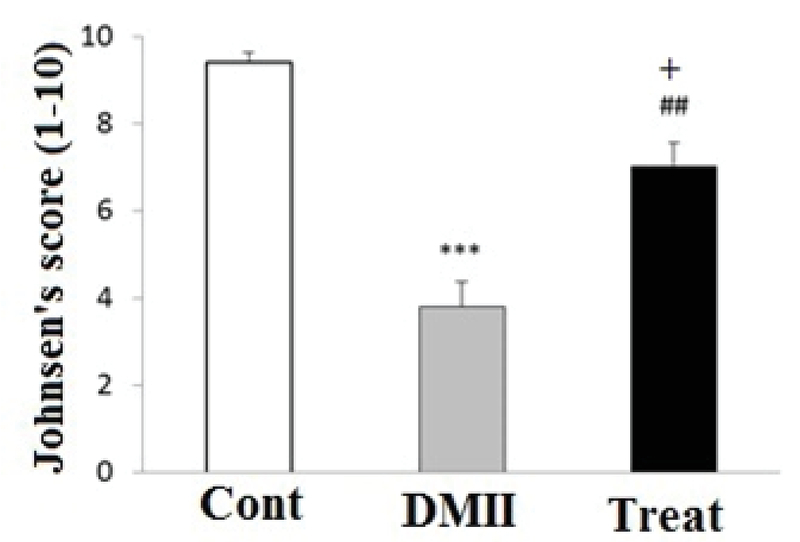
Effects of application of Crocin on Johnsen's score in testis tissue of control and experimental groups of rats. Data are presented as mean ± SE. ${}^{***}$p< 0.001 compared to Cont group; +p< 0.05 compared to Cont group; and ${}^{\#\#}$p< 0.01 compared to DMII group; Cont: Control group; DMII: Diabetic group; and treat: Treatment group.

**Figure 6:**
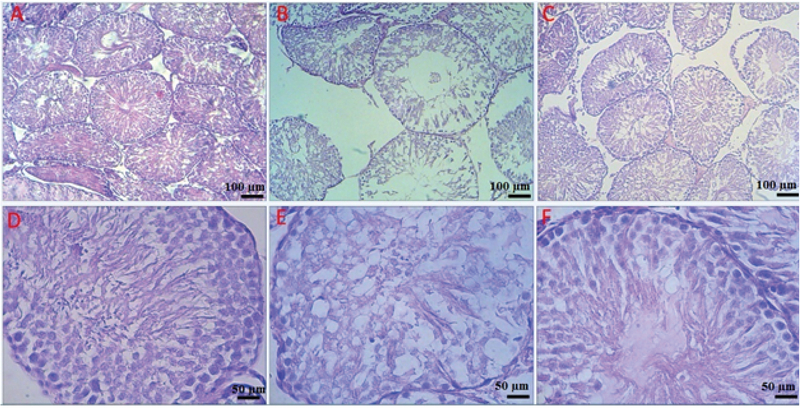
The effects of application of Crocin on testis histopathological changes induced by HFD/STZ. Photographs indicated several seminiferous tubules in rats stained with H&E in (A) control, (B) diabetic; and (C) treatment groups; (Scale bar= 100 µm, 100X). Diabetic rats treated with RJ showed improvement in seminiferous tubular structure. Image of (D), (E), and (F) indicated magnified seminiferous tubules in control, diabetic, and treatment groups, respectively; (Scale bar= 50 µm, 400X).

Mean Johnsen's score of the group DMII was significantly lower as compared to the control group (Figure 5, p≤ 0.001). Mean Johnsen's score of treatment group significantly increased as compared with the Johnsen's score of DMII group (Figure 5, p= 0.001). Moreover, mean Johnsen's score of treatment group was significantly lower than the control group (Figure 5, p= 0.011). Our histopathological results identified that the testis morphology was histologically normal in the control normal group (Figure 6). However, the pathological changes of seminiferous tubules in the diabetic group showed degeneration with disorganized epithelium in DMII group (Figure 6). Moreover, other typical changes such as depletion of germ cells, declined layers of seminiferous epithelium could also be seen in the diabetic group. Furthermore, application of Crocin could improve and reverse the STZ-induced testopathy in the treatment rats as compared with the DMII rats (Figure 6).

## 4. Discussion

The present study identified that application of HFD and STZ induced abnormal histological structure alterations in testis tissue. Furthermore, the present results demonstrated the protective potential of Crocin against hyperglycemia-mediated dysfunction of testis tissue in DMII rats. It is reported that administration of STZ can induce DM via reduction of the beta cells mass and subsequently insulin secretion, which are leading to prolonged hyperglycemia (32). In the current study, the DMII group had a drastically higher serum glucose and cholesterol levels as compared with the control group. Application of Crocin caused a significant decrease in serum glucose in treatment group. It is likely that the anti-hyperglycemic effects of Crocin are related to its inhibitory effects on the insulin resistance or its stimulatory effects on the secretion of insulin in diabetic rats (33). Moreover, our data identified that the administration of Crocin to the diabetic rats decreased serum cholesterol levels. In the elucidation of the hypolipidemic mechanism of Crocin, it may act by decreasing the absorption of fat and cholesterol by suppressing pancreatic lipase function (34). Several reports have demonstrated that the inhibiting effects of Crocin on cholesterol level may be related to the reduction of the absorption of fat or its effects on disruption of the bile acid enterohepatic circulation (35). Several reports have shown that the reproductive functions are markedly affected by DM, which can lead to reduced fertility (36). For example, it is reported that the application of the STZ result in abnormal anatomical alterations in the seminiferous tubule cells such as spermatogenic, Sertoli, and Leydig cells (37). Our results have shown that the induction of DMII leads to abnormal structural changes in the rats. The interactions of all cells such as Sertoli, peritubular, and Leydig cells are important for monitoring the normal spermatogenic function within the seminiferous tubules (38). In agreement with these studies, our data represent that DM induces abnormal changes in architect of testis and also decreases sperm count and the total size of testis in the DMII group compared with the control group. Slegtenhorst-Eegdeman *et al*. reported that the size of the testis is related to sperm production and the germinal cell numbers (39). The present study was intended to investigate the protective actions of Crocin on diabetic-induced abnormality of testis tissue. It appeared from the results of the present study that the application of Crocin significantly increased the number of total and normal sperms and also the weight of testes in treatment group as compared with the DMII group. Our data have also shown that the application of Crocin improves the pathological changes in the testis of treatment rats as compared with the DMII rats.

## 5. Conclusion

Our findings concluded that diabetes mellitus in male rats caused pathological changes in testis, and that Crocin treatment improved these deficits by increasing the number of sperms and sperm production.

##  Conflict of Interest

The authors declare that there is no conflict of interest in this study.
